# Supplementary material for: Sequence Analysis and Structural Predictions of Lipid Transfer Bridges in the Repeating Beta Groove (RBG) Superfamily Reveal Past and Present Domain Variations Affecting Form, Function and Interactions of VPS13, ATG2, SHIP164, Hobbit and Tweek
Source: Contact (Thousand Oaks). 2022 Nov 21;5:25152564221134328. doi: 10.1177/25152564221134328 (PMC7613979; doi:10.1177/25152564221134328)
Supplement: sj-docx-1-ctc-10.1177_25152564221134328 - Supplemental material for Sequence Analysis and Structural Predictions of Lipid Transfer Bridges in the Repeating Beta Groove (RBG) Superfamily Reveal Past and Present Domain Variations Affecting Form, Function and Interactions of VPS13, ATG2, SHIP164, Hobbi [file sj-docx-1-ctc-10.1177_25152564221134328.docx]

# **Supplementary Information for:** Structural and sequence analysis of lipid transfer bridges in the repeating beta groove (RBG) superfamily VPS13, ATG2, SHIP164, Hobbit and Tweek reveals past and present domain variations affecting form function and interactions.

**Supplementary Figures**

**Supplementary Figure 1.** RBG domain cluster map identifies distance between duplicates of RBG5 and RBG6.


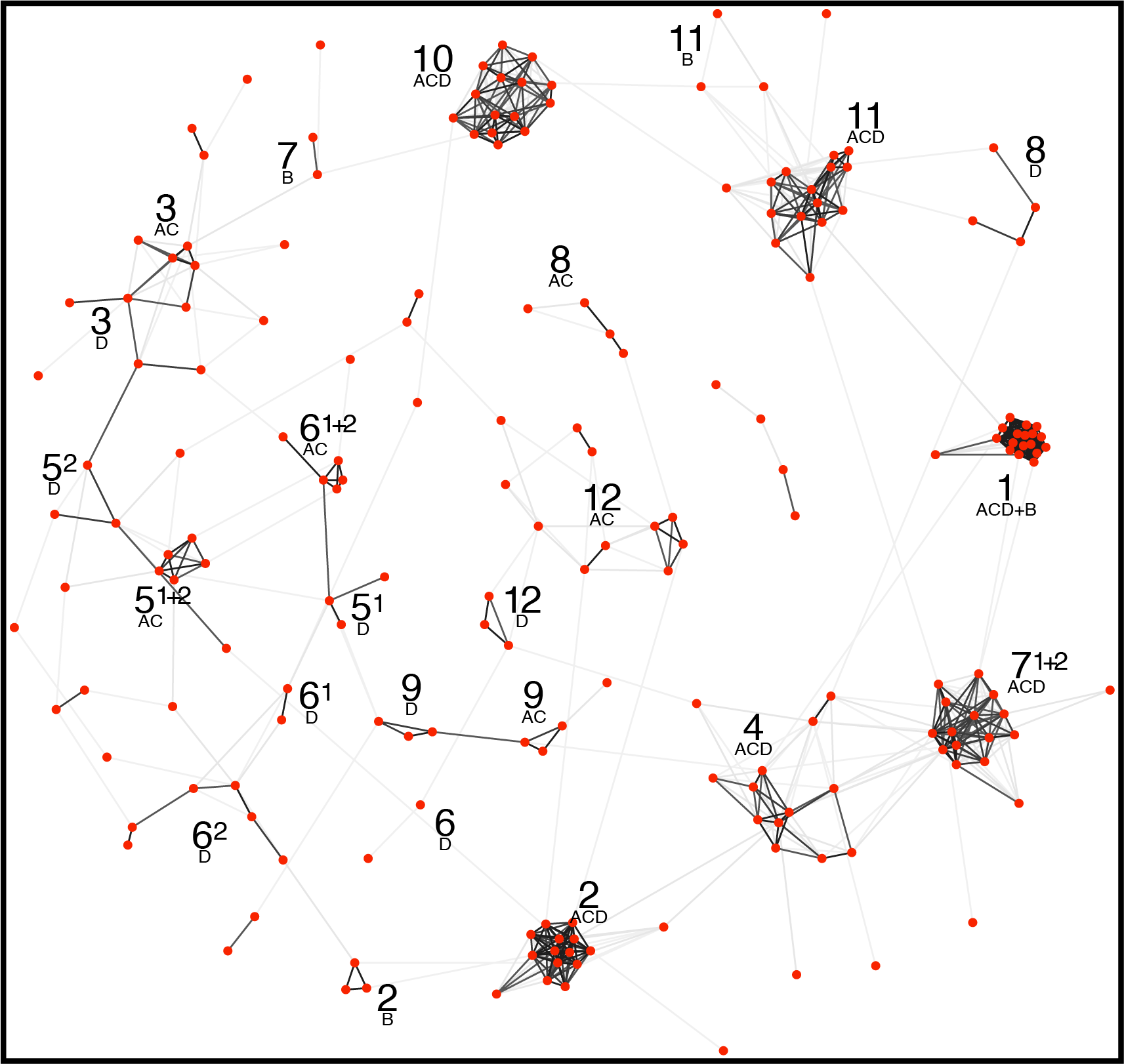


Cluster map made in CLANS of 162 linked RBG domains from VPS13 proteins from 7 diverse eukaryotes (see Methods). Stronger homologies, shown by short and thick lines, is confined to orthologous domains. All RBG7s clustered tightly, including RBG7C/D^1/2^. By comparison, RBG5C^1/2^ and RBG6C^1/2^ form two clustered groups separate from RBG5D^1/2^ and RBG6D^1/2^ respectively, and in both RBG5 and RBG6 D^1^ is only indirectly or weakly connected with D^2^. This suggests that the duplication in VPS13D was a separate earlier event from that in VPS13C.

**Supplementary Figure 2.** VPS13B homologs are spread across eukaryotic evolution


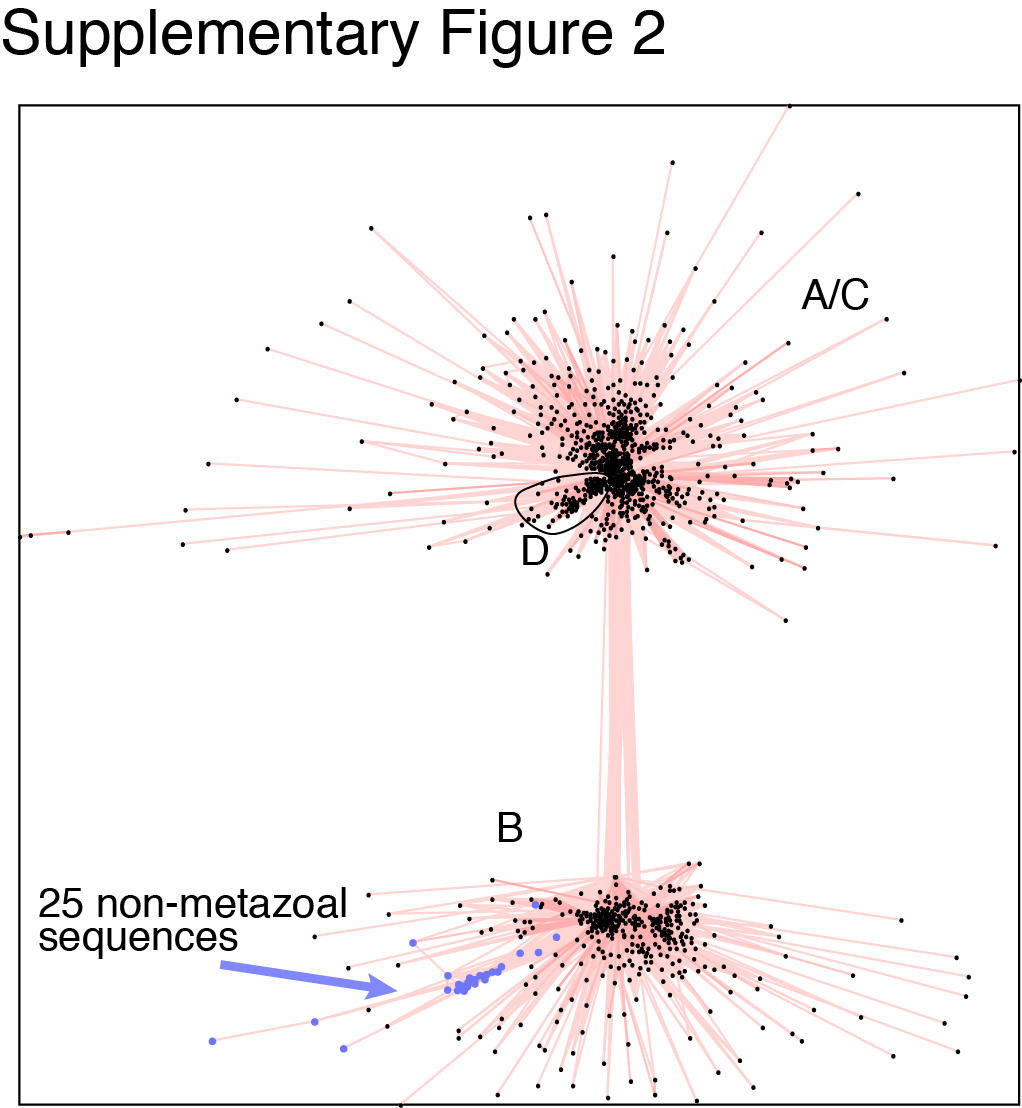


Cluster map of 1186 VPS13 proteins constructed in CLANS as described in Methods. VPS13B and close homologs clustered separately from VPS13A/C/D, in which VPS13D form one segment. The VPS13B cluster contains 25 protist proteins (blue): Albugo, A0A024G1K2; Aphanomyces, A0A397D2M9, A0A418DCD9, A0A397ERH8, A0A024TE03, A0A6A4ZLW7; Bremia, A0A484ECX3; Chara, A0A388LFT4; Chromera, A0A0G4FFP3; Globisporangium, K3X2H6; Guillardia, A0A7S4JEF6; Hanusia, A0A7S0E6H4; Hemiselmis, A0A7S0TJS0; Hondaea, A0A2R5GB42; Hyaloperonospora, M4BW50; Klebsormidium, A0A1Y1HLB4; labyrinthulid, A0A7S2S870; Phytophthora, A0A0W8B040, A0A081AUX5, A0A0W8D6K5, H3H1A7, A0A3F2RKP7; Pythium, A0A2D4C9P6; Thecamonas, A0A0L0DHF4; Vitrella, A0A0G4GLM3.

**Supplementary Figure 3.** RBG domain 5 of VPS13X from *A. thaliana* has 7 strands.


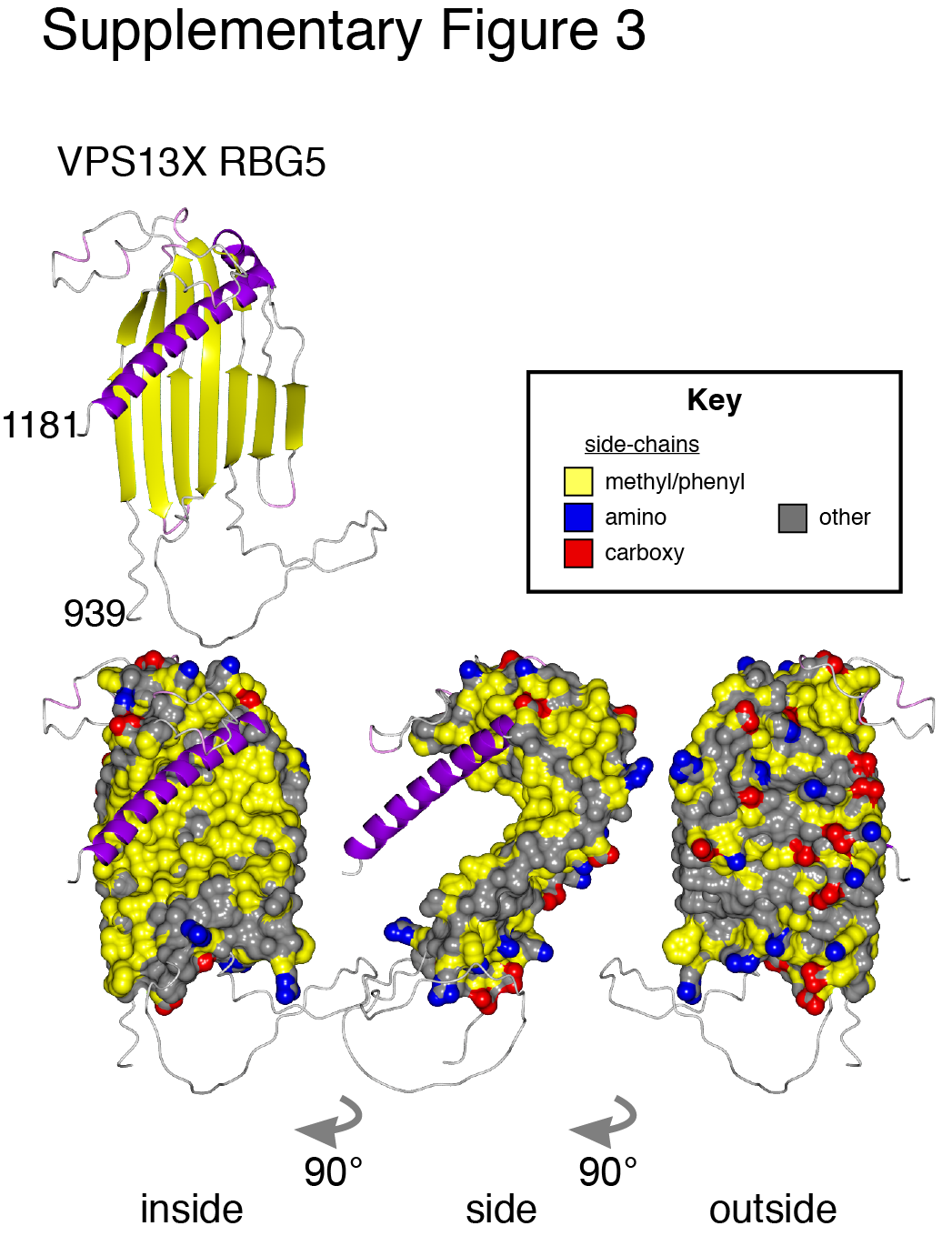


Residues 939-1181 of VPS13X from *A. thaliana* At3g50380 were modelled by ColabFold, and visualized both in ribbon form showing the inside of the groove (top, strands – yellow, helices – purple) and with the surface of the sheet colored according to the YRB scheme (see key (Hagemans *et al.*, 2015)) indicating its hydrophobicity, together with two other orientations showing the side and hydrophilic outside of the groove. pLDDT of the model (excluding inserted loops) was 83% over 174 residues.

**Supplementary Figure 4.** Amphipathic helices at the extreme C-termini of RBG multimers


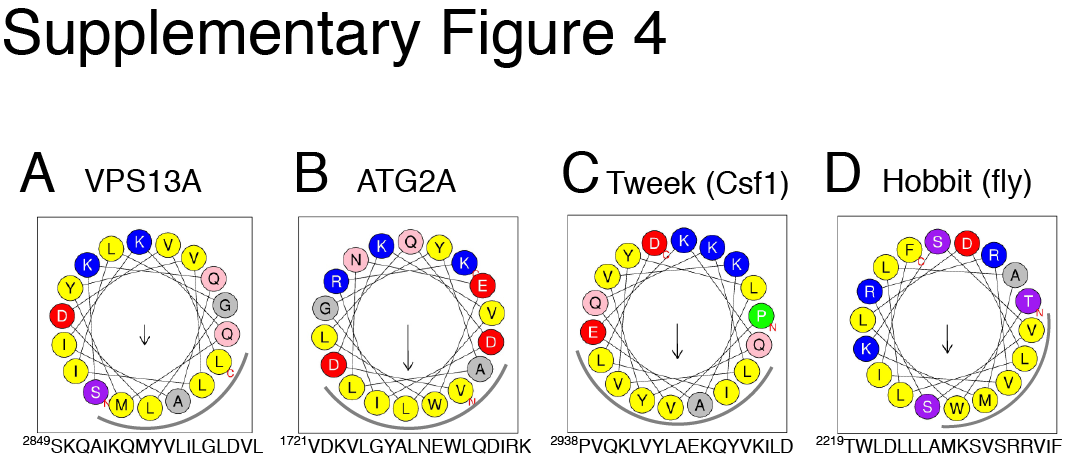


A–D Helical wheel projections (from Heliquest) of the indicated residues immediately following the final RBG domain of VSP13A (human), ATG2A (human), yeast Tweek (Csf1) and fly Hobbit respectively. Images created by Heliquest, with hydrophobic face indicated by an arc.

**Supplementary Figure 5.** Conserved residues on the concave (outside) face of domains near the central regions of RBG proteins


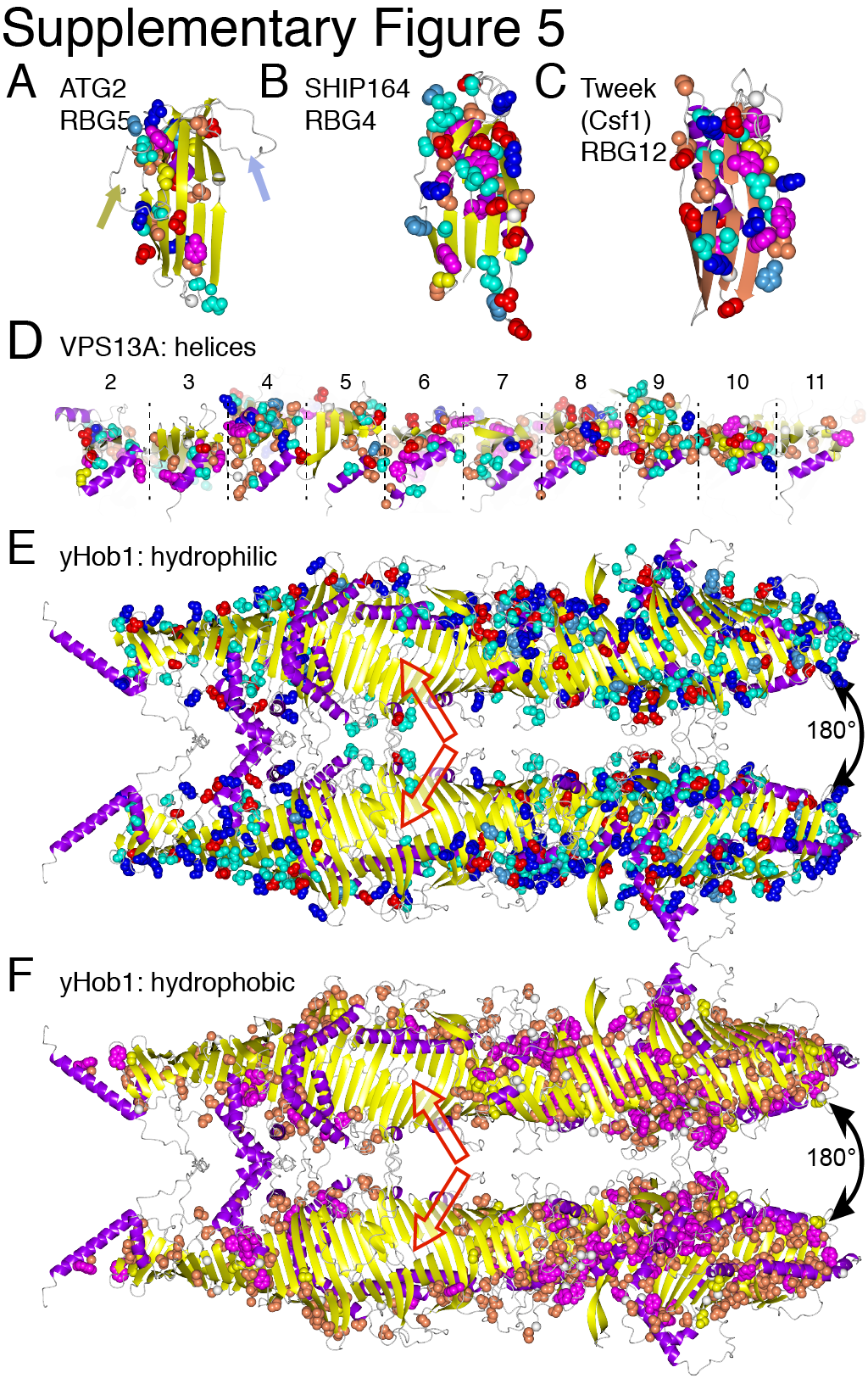


A–C: Concave (outside) faces of: A. ATG2 RBG5, B. SHIP164 RBG4, C. yeast Tweek (Csf1) RBG12. Identification and coloring of conserved residues (all), colored as in Figure 7. ATG2 RBG5 has both a loop between strands 3 and 4 (light blue arrow) and a bulge onto the convex surface between the two halves of strand 4 (gold arrow). D. 10 central RBG domains from VPS13A rotated to view helices and adjacent sheet, with all conserved residues shown as spheres, coloring as in Figure 7, same models as Figure 8B–D. Note that conserved residues pack the interface between helix and sheet in half of the RBG domains: RBG3/6/8/9/10. E and F: Yeast Hob1 (aka Fmp27) showing conserved hydrophilic (E) and hydrophobic (F) residues, as in Figure 8C/D, with views rotated by 180° in both cases. Three pairs of antiparallel helices have been removed. Open arrows indicate a region with few conserved residues 559-892 (strands 4/5 of RBG4 and all of RBG5). This differs from the orthologous region of human Hobbit, where RBG5 shows wide homology, for example to its plant ortholog (Figure 3E).

**Supplementary Data File 1: Boundaries of RBG and other domains**

Boundaries of all RBG domains and non-standard accessory domains in selected proteins from five RBG protein families. The C-terminal boundaries include the initial portion only (12-20 aa) of the 6^th^ element (loop) which usually includes the start of the helix.

**VPS13 (22 proteins in 8 model organisms)**

human–A (3174 aa):– RBG domains (12): 1-145, 146-309, 480-609, 642-796, 877-1020, 1049-1181, 1205-1342, 1390-1527, 1549-1672, 1720-1855, 2518-2723 (omit WWE), 2762-2845; accessory domain: WWE (2553-2624)

human–B (4022 aa):– RBG domains (13): 1-149, 150-284, 386-612, 637-819, 838-966, 1064-1232, 1320-1519, 1546-1750, , 2116-2269, 2473-2612, 3380-3549, 3581-3639; accessory domain: β-sandwich (2295-2472)

human–C (3753 aa):– RBG domains (15): 1-191, 192-350, 524-658, 685-835, 906-1049, 1076-1214, 1236-1374, 1429-1558, 1589-1729, 1751-1889, 1940-2068, 2100-2223, 2274-2401, 3081-3282 (omit WWE), 3321-3402; accessory domain: WWE (3116-3181)

human–D (4388 aa):– RBG domains (15): 1-149, 150-300, 493-650, 704-904, 949-1130, 1158-1316, 1367-1556, 1611-1837, 1868-1990, 2017-2258, 2281-2418, 2461-2597, 2701-2814, 3820-3949, 3981-4050; accessory domains: UBA (2635-2676), Ricin (3569-3774)

fly–AC (3321 aa):– RBG domains (12): 1-149, 150-300, 476-620, 647-809, 899-1042, 1093-1224, 1245-1386, 1436-1581, 1623-1743, 1804-1934, 2663-2863, 2897-2977; accessory domain: WWE (2695-2769)

fly–B (3731 aa):– RBG domains (13): 1-149, 150-300, 338-536, 576-742, 759-865, 973-1120, 1182-1327, 1355-1534, 1685-1804, 1836-1943, 2168-2309, 3125-3278, 3316-3370; accessory domain: Ricin (2010-2167)

fly–D (3919 aa):– RBG domains (15): 1-149, 150-300, 475-612, 649-847, 874-986, 1007-1239, 1286-1422, 1456-1641, 1665-1789, 1814-1969, 2006-2140, 2164-2275, 2343-2471, 3357-3491, 3533-3592; accessory domains: UBA (2293-2338), Ricin (3120-3305)

worm–AC (3212 aa):– RBG domains (12): 1-147, 148-303, 488-594, 632-779, 842-966, 1038-1166, 1200-1332, 1381-1523, 1559-1679, 1748-1872, 2562-2765, 2800-2850; accessory domain: WWE (2594-2666)

worm–D (3312 aa):– RBG domains (11): 1-153, 154-301, 562-709, 733-976, 1001-1151, 1174-1344, 1373-1510, 1574-1716, 1745-1878, 2670-2857, 2888-2962; accessory domain: Ricin (2543-2647)

*Trichoplax*–AC (1561 aa):– RBG domains (/): 1-115, [RBG 2-9], 127-258, 899-1101, 1145-1222; accessory domain: WWE (936-1002)

*Trichoplax*–D (4149 aa):– RBG domains (15): 1-150, 151-302, 514-655, 694-890, 945-1096, 1119-1302, 1371-1520, 1546-1710, 1745-1863, 1891-2063, 2092-2226, 2273-2410, 2509-2657, 3576-3624, 3739-3809; accessory domains: UBA (2430-2477), Ricin (3318-3513)

*S. cerevisiae* (3144 aa):– RBG domains (12): 1-157, 158-314, 505-645, 684-841, 890-1035, 1063-1184, 1210-1345, 1395-1525, 1560-1670, 1738-1855, 2579-2708, 2741-2819

*S. pombe–i* (3004 aa):– RBG domains (12): 1-149, 150-297, 462-587, 625-769, 811-949, 971-1090, 1116-1249, 1289-1416, 1450-1558, 1619-1736, 2438-2566, 2601-2676

*S. pombe–ii* (3071 aa):– RBG domains (12): 1-149, 150-306, 484-623, 662-815, 869-1008, 1036-1155, 1182-1315, 1352-1481, 1512-1623, 1678-1800, 2500-2631, 2662-2739

*Capsaspora*–AC (6160 aa):– RBG domains (13): 1-153, 154-401, 690-891, 929-1049, 1060-1420, 1440-2180, 2230-2370, 2450-2650, 2740-3060, 3110-3282, 3374-3496, 5030-5251, 5350-5507; accessory domains: helical (1199-2128), alpha/beta (unknown) (2845-3002)

*Capsaspora*–B (4668 aa):– RBG domains (13): 1-194, 195-400, 538-870, 902-1118, 1143-1312, 1380-1575, 1647-1870, 1937-2166, 2303-2460, 2518-2710, 2715-2890, 3940-4120, 4166-4222; accessory domains: helical (1675-1929), helical (2203-2298), helical (4327-4415)

*Capsaspora*–D (4768 aa):– RBG domains (15): 1-158, 159-307, 550-683, 719-1014, 1083-1246, 1311-1463, 1524-1654, 1736-1878, 1954-2119, 2148-2311, 2462-2602, 2726-2890, 3014-3138, 4164-4291, 4441-4514; accessory domains: helical (2936-3002), Ricin (3865-4090)

*Capsaspora*–X (3708 aa):– RBG domains (12): 1-156, 157-314, 540-670, 712-894, 972-1118, 1190-1319, 1376-1550, 1613-1746, 1823-1977, 2063-2191, 3124-3283, 3390-3538

*Arabidopsis*–S (3464 aa):– RBG domains (12): 1-149, 150-300, 464-598, 630-787, 802-952, 1055-1195, 1223-1376, 1429-1529, 1556-1731, 1748-1866, 2791-2941, 3017-3091

*Arabidopsis*–M1 (4219 aa):– RBG domains (12): 1-149, 150-300, 477-598, 633-780, 942-1159, 1197-1329, 1351-1488, 1533-1712, 1863-1974, 2395-2512, 3436-3565, 3584-3654; accessory domains: PH (801-909), beta-helix (1720-1860), beta-tripod (1983-2152), beta-tripod (2210-2374), C2 (2607-2765), beta-tripod (3972-4220)

*Arabidopsis*–M2 (4146 aa):– RBG domains (12): 1-169, 170-300, 533-657, 696-839, 1010-1238, 1281-1415, 1444-1573, 1615-1766, 1931-2038, 2440-2560, 3562-3702, 3711-3781; accessory domains: PH (874-989), beta-helix (1790-1930), beta-tripod (2039-2190), beta-tripod (2258-2420), C2 (2660-2820)

*Arabidopsis*–X (3125 aa):– RBG domains (9): 1-157, 158-314, 465-680, 730-900, 939-1181, 1207-1397, 1611-1742, 2558-2693, 2702-2776; accessory domains: Ricin (1428-1583), helices (partly amphiphathic) (2996-3125)

**ATG2**

human-B (2078 aa):- RBG domains (8): 1-132,163-335,505-701,738-930,1049-1218,1232-1363,1544-1726,1806-1869

*Arabidopsis* (1892 aa):- RBG domains (8): 1-120,172-325,457-639,661-869,895-1046,1062-1200,1428-1578,1612-1680

yeast (1592 aa):- RBG domains (8): 1-118,176-338,373-462,492-600,644-756,783-904,1025-1245,1262-1329

**SHIP164**

human (UHRF1BP1) (1464 aa):- RBG domains (6): 1-105,118-257,349-547,569-831,855-1300,1311-1370

*Arabidopsis* (1199 aa):- RBG domains (6): 1-126,127-290,315-466,481-654,688-843,902-1068

**Hobbit**

Human KIAA0100 (Hobbit, 2235 aa):- 1-134, 135-247, 276-393, 415-560, 568-685, 724-846, 869-1113, 1134-1293, 1318-1471, 1574-1774, 1907-2028, 2113-2166; accessory domain: paired helices 884-959, (1429-1444 – *i.e*. missing), 1781-1904

yeast Hob1 (Fmp27, 2628 aa):- RBG domains (12): 1-125, 125-225, 274-426, 467-593, 655-847, 893-1015, 1053-1403, 1429-1651, 1680-1985, 2070-2239, 2340-2470, 2522-2579; accessory domain: paired helices 1067-1234, 1835-1960, 2244-2339

**Tweek**

yeast Csf1:- RBG domains (17): 61-154, 226-448, 468-607, 627-768, 841-972, 993-1236 (omit loop with 2 helices 1081-1202), 1265-1389, 1534-1610, 1627-1745, 1778-1884, 1920-2035, 2077-2193, 2238-2320, 2354-2472, 2510-2671, 2712-2852, 2891-2922; accessory domains: helical bundle 1410-1520.

**Movies**

RBG domains only from human VPS13A (N-terminus to the left) showing: Movie 1 back bone only, Movie 2 conserved hydrophilic residues, Movie 3 hydrophobic residues.
