# Supplementary material for: Sequence Analysis and Structural Predictions of Lipid Transfer Bridges in the Repeating Beta Groove (RBG) Superfamily Reveal Past and Present Domain Variations Affecting Form, Function and Interactions of VPS13, ATG2, SHIP164, Hobbit and Tweek
Source: Contact (Thousand Oaks). Author manuscript; Available in PMC 2022 Dec 22. (PMC7613979; doi:10.1177/25152564221134328)
Supplement: Supplementary file 1 [file EMS158510-supplement-Supplementary_Information.pdf]

Supplemental Material

Supplemental material for this article is available online.

<u>Supplementary Information for:</u> Structural and sequence analysis of lipid transfer bridges in the repeating beta groove (RBG) superfamily VPS13, ATG2, SHIP164, Hobbit and Tweek reveals past and present domain variations affecting form function and interactions.

<u>Supplementary Figures</u>

Supplementary Figure 1. RBG domain cluster map identifies distance between duplicates of RBG5 and RBG6.

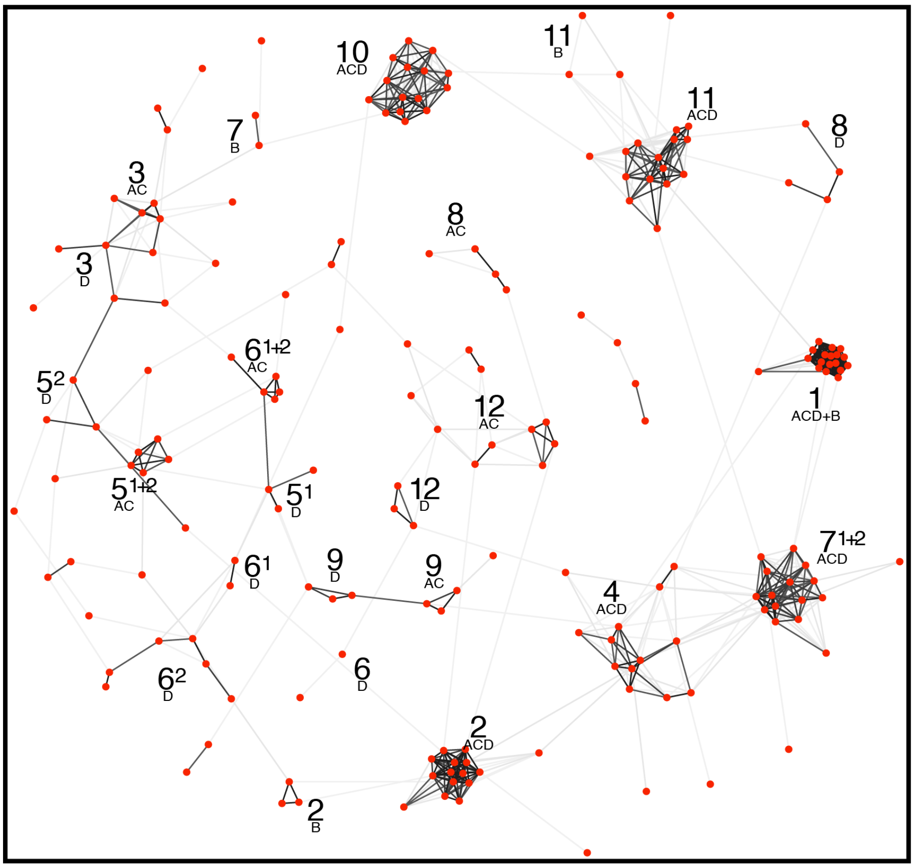

Cluster map made in CLANS of 162 linked RBG domains from VPS13 proteins from 7 diverse eukaryotes (see Methods). Stronger homologies, shown by short and thick lines, is confined to orthologous domains. All RBG7s clustered tightly, including RBG7C/D$^{1/2}$. By comparison, RBG5C$^{1/2}$ and RBG6C$^{1/2}$ form two clustered groups separate from RBG5D$^{1/2}$ and RBG6D$^{1/2}$ respectively, and in both RBG5 and RBG6 D^{1} is only indirectly or weakly connected with D^{2}. This suggests that the duplication in VPS13D was a separate earlier event from that in VPS13C.

Supplementary Figure 2. VPS13B homologs are spread across eukaryotic evolution

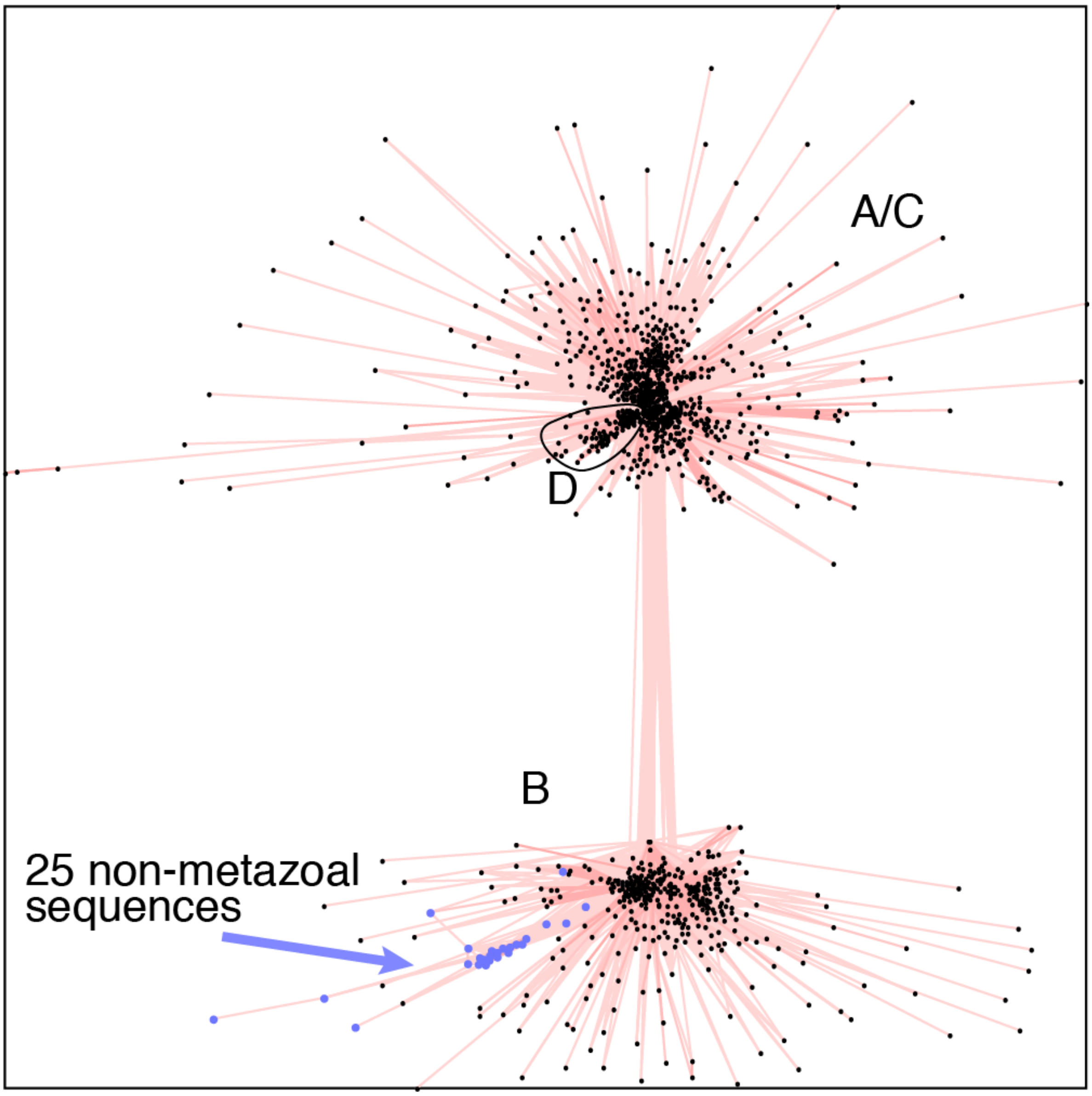

Cluster map of 1186 VPS13 proteins constructed in CLANS as described in Methods. VPS13B and close homologs clustered separately from VPS13A/C/D, in which VPS13D form one segment. The VPS13B cluster contains 25 protist proteins (blue): Albugo, A0A024G1K2; Aphanomyces, A0A397D2M9, A0A418DCD9, A0A397ERH8, A0A024TE03, A0A6A4ZLW7; Bremia, A0A484ECX3; Chara, A0A388LFT4; Chromera, A0A0G4FFP3; Globisporangium, K3X2H6; Guillardia, A0A7S4JEF6; Hanusia, A0A7S0E6H4; Hemiselmis, A0A7S0TJS0; Hondaea, A0A2R5GB42; Hyaloperonospora, M4BW50; Klebsormidium, A0A1Y1HLB4; labyrinthulid, A0A7S2S870; Phytophthora, A0A0W8B040, A0A081AUX5, A0A0W8D6K5, H3H1A7, A0A3F2RKP7; Pythium, A0A2D4C9P6; Thecamonas, A0A0L0DHF4; Vitrella, A0A0G4GLM3.

Supplementary Figure 3. RBG domain 5 of VPS13X from *A. thaliana* has 7 strands.

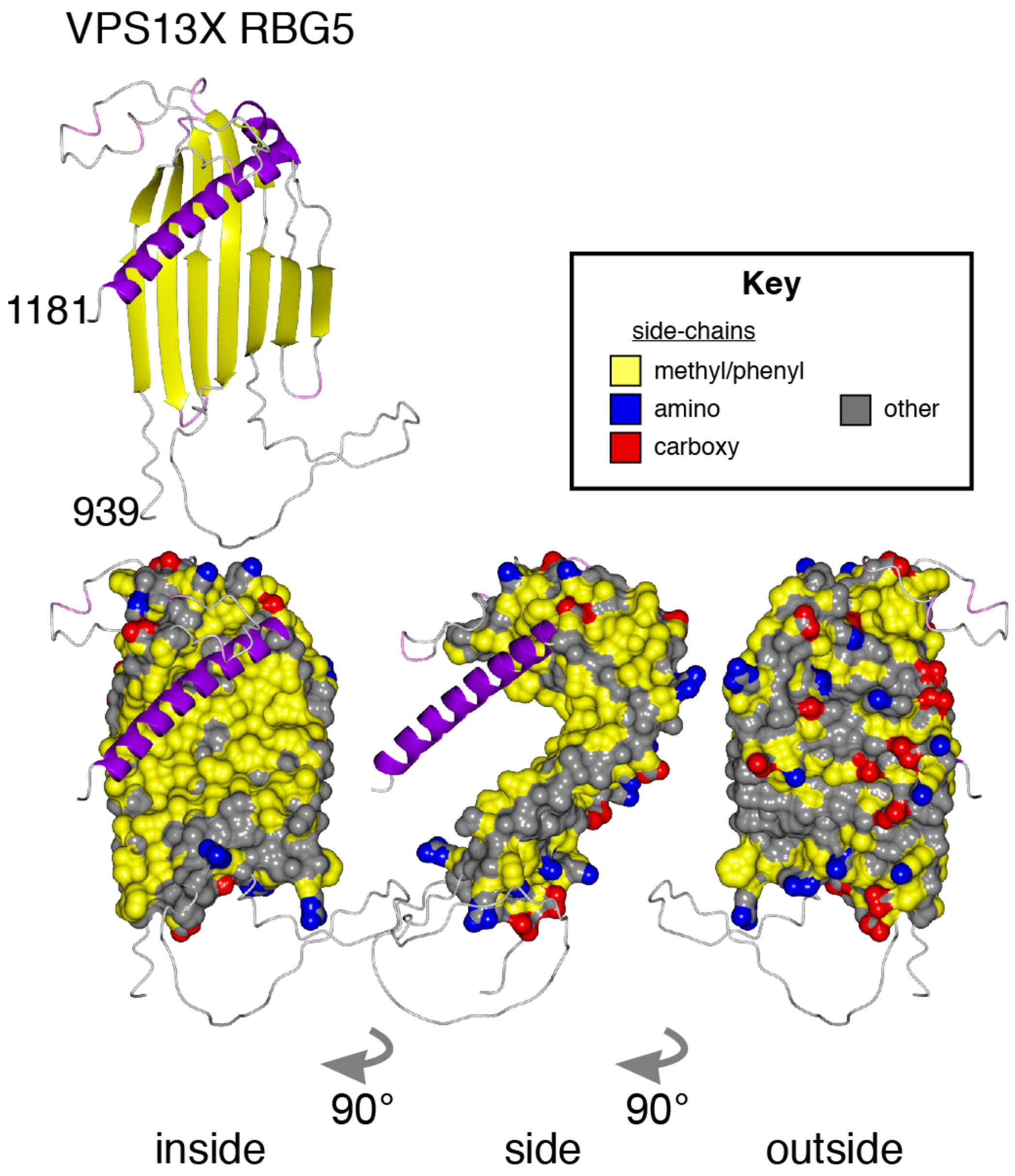

Residues 939-1181 of VPS13X from *A. thaliana* At3g50380 were modelled by ColabFold, and visualized both in ribbon form showing the inside of the groove (top, strands – yellow, helices – purple) and with the surface of the sheet colored according to the YRB scheme (see key (Hagemans *et al.*, 2015)) indicating its hydrophobicity, together with two other orientations showing the side and hydrophilic outside of the groove. pLDDT of the model (excluding inserted loops) was 83% over 174 residues.

Supplementary Figure 4. Amphipathic helices at the extreme C-termini of RBG multimers

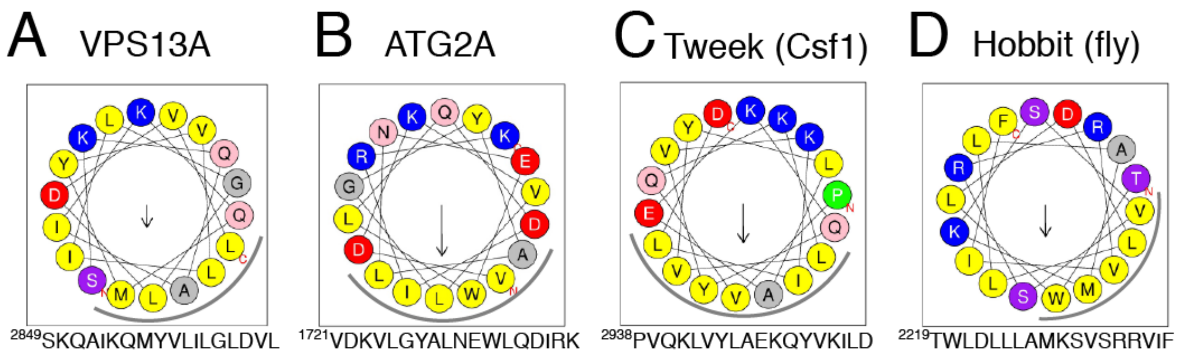

A–D Helical wheel projections (from Heliquest) of the indicated residues immediately following the final RBG domain of VSP13A (human), ATG2A (human), yeast Tweek (Csf1) and fly Hobbit respectively. Images created by Heliquest, with hydrophobic face indicated by an arc.

Supplementary Figure 5. Conserved residues on the concave (outside) face of domains near the central regions of RBG proteins

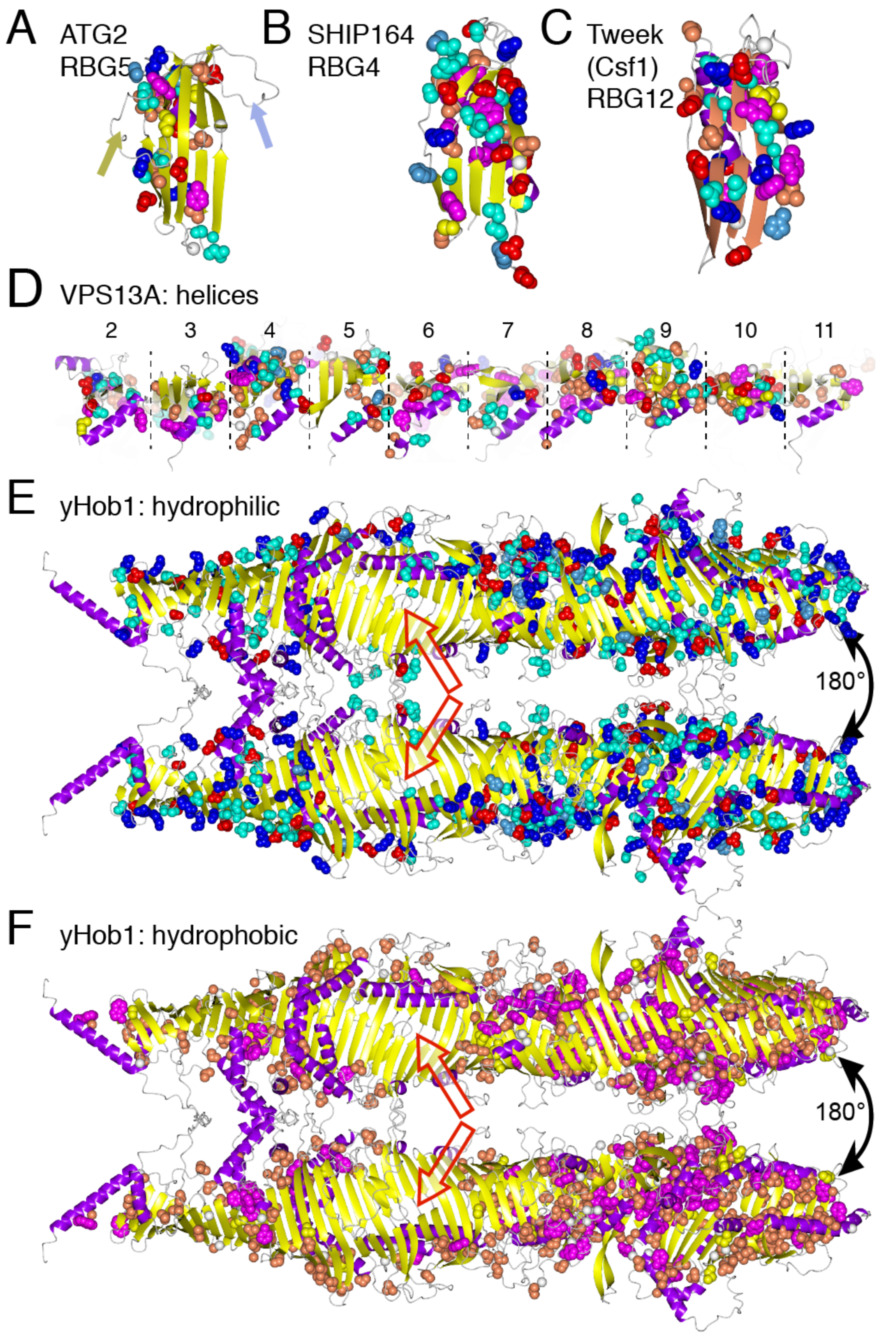

A–C: Concave (outside) faces of: A. ATG2 RBG5, B. SHIP164 RBG4, C. yeast Tweek (Csf1) RBG12. Identification and coloring of conserved residues (all), colored as in Figure 7. ATG2 RBG5 has both a loop between strands 3 and 4 (light blue arrow) and a bulge onto the convex surface between the two halves of strand 4 (gold arrow). D. 10 central RBG domains from VPS13A rotated to view helices and adjacent sheet, with all conserved residues shown as spheres, coloring as in Figure 7, same models as Figure 8B–D. Note that

conserved residues pack the interface between helix and sheet in half of the RBG domains: RBG3/6/8/9/10. E and F: Yeast Hob1 (aka Fmp27) showing conserved hydrophilic (E) and hydrophobic (F) residues, as in Figure 8C/D, with views rotated by 180° in both cases. Three pairs of antiparallel helices have been removed. Open arrows indicate a region with few conserved residues 559-892 (strands 4/5 of RBG4 and all of RBG5). This differs from the orthologous region of human Hobbit, where RBG5 shows wide homology, for example to its plant ortholog (Figure 3E).

Supplementary Data File 1: Boundaries of RBG and other domains

Boundaries of all RBG domains and non-standard accessory domains in selected proteins from five RBG protein families. The C-terminal boundaries include the initial portion only (12-20 aa) of the 6th element (loop) which usually includes the start of the helix.

VPS13 (22 proteins in 8 model organisms)

human–A (3174 aa):– RBG domains (12): 1-145, 146-309, 480-609, 642-796, 877-1020, 1049-1181, 1205-1342, 1390-1527, 1549-1672, 1720-1855, 2518-2723 (omit WWE), 2762-2845; accessory domain: WWE (2553-2624)

human–B (4022 aa):– RBG domains (13): 1-149, 150-284, 386-612, 637-819, 838-966, 1064-1232, 1320-1519, 1546-1750, , 2116-2269, 2473-2612, 3380-3549, 3581-3639; accessory domain: β-sandwich (2295-2472)

human–C (3753 aa):– RBG domains (15): 1-191, 192-350, 524-658, 685-835, 906-1049, 1076-1214, 1236-1374, 1429-1558, 1589-1729, 1751-1889, 1940-2068, 2100-2223, 2274-2401, 3081-3282 (omit WWE), 3321-3402; accessory domain: WWE (3116-3181)

human–D (4388 aa):– RBG domains (15): 1-149, 150-300, 493-650, 704-904, 949-1130, 1158-1316, 1367-1556, 1611-1837, 1868-1990, 2017-2258, 2281-2418, 2461-

2597, 2701-2814, 3820-3949, 3981-4050; accessory domains: UBA (2635-2676), Ricin (3569-3774)

fly–AC (3321 aa):– RBG domains (12): 1-149, 150-300, 476-620, 647-809, 899-1042, 1093-1224, 1245-1386, 1436-1581, 1623-1743, 1804-1934, 2663-2863, 2897-2977; accessory domain: WWE (2695-2769)

fly–B (3731 aa):– RBG domains (13): 1-149, 150-300, 338-536, 576-742, 759-865, 973-1120, 1182-1327, 1355-1534, 1685-1804, 1836-1943, 2168-2309, 3125-3278, 3316-3370; accessory domain: Ricin (2010-2167)

fly–D (3919 aa):– RBG domains (15): 1-149, 150-300, 475-612, 649-847, 874-986, 1007-1239, 1286-1422, 1456-1641, 1665-1789, 1814-1969, 2006-2140, 2164-2275, 2343-2471, 3357-3491, 3533-3592; accessory domains: UBA (2293-2338), Ricin (3120-3305)

worm–AC (3212 aa):– RBG domains (12): 1-147, 148-303, 488-594, 632-779, 842-966, 1038-1166, 1200-1332, 1381-1523, 1559-1679, 1748-1872, 2562-2765, 2800-2850; accessory domain: WWE (2594-2666)

worm–D (3312 aa):– RBG domains (11): 1-153, 154-301, 562-709, 733-976, 1001-1151, 1174-1344, 1373-1510, 1574-1716, 1745-1878, 2670-2857, 2888-2962; accessory domain: Ricin (2543-2647)

Trichoplax–AC (1561 aa):– RBG domains (/): 1-115, [RBG 2-9], 127-258, 899-1101, 1145-1222; accessory domain: WWE (936-1002)

Trichoplax–D (4149 aa):– RBG domains (15): 1-150, 151-302, 514-655, 694-890, 945-1096, 1119-1302, 1371-1520, 1546-1710, 1745-1863, 1891-2063, 2092-2226, 2273-2410, 2509-2657, 3576-3624, 3739-3809; accessory domains: UBA (2430-2477), Ricin (3318-3513)

S. cerevisiae (3144 aa):– RBG domains (12): 1-157, 158-314, 505-645, 684-841, 890-1035, 1063-1184, 1210-1345, 1395-1525, 1560-1670, 1738-1855, 2579-2708, 2741-2819

S. pombe–i (3004 aa):– RBG domains (12): 1-149, 150-297, 462-587, 625-769, 811-949, 971-1090, 1116-1249, 1289-1416, 1450-1558, 1619-1736, 2438-2566, 2601-2676

S. pombe–ii (3071 aa):– RBG domains (12): 1-149, 150-306, 484-623, 662-815, 869-1008, 1036-1155, 1182-1315, 1352-1481, 1512-1623, 1678-1800, 2500-2631, 2662-2739

Capsaspora–AC (6160 aa):– RBG domains (13): 1-153, 154-401, 690-891, 929-1049, 1060-1420, 1440-2180, 2230-2370, 2450-2650, 2740-3060, 3110-3282, 3374-3496, 5030-5251, 5350-5507; accessory domains: helical (1199-2128), alpha/beta (unknown) (2845-3002)

Capsaspora–B (4668 aa):– RBG domains (13): 1-194, 195-400, 538-870, 902-1118, 1143-1312, 1380-1575, 1647-1870, 1937-2166, 2303-2460, 2518-2710, 2715-2890, 3940-4120, 4166-4222; accessory domains: helical (1675-1929), helical (2203-2298), helical (4327-4415)

Capsaspora–D (4768 aa):– RBG domains (15): 1-158, 159-307, 550-683, 719-1014, 1083-1246, 1311-1463, 1524-1654, 1736-1878, 1954-2119, 2148-2311, 2462-2602, 2726-2890, 3014-3138, 4164-4291, 4441-4514; accessory domains: helical (2936-3002), Ricin (3865-4090)

Capsaspora–X (3708 aa):– RBG domains (12): 1-156, 157-314, 540-670, 712-894, 972-1118, 1190-1319, 1376-1550, 1613-1746, 1823-1977, 2063-2191, 3124-3283, 3390-3538

Arabidopsis–S (3464 aa):– RBG domains (12): 1-149, 150-300, 464-598, 630-787, 802-952, 1055-1195, 1223-1376, 1429-1529, 1556-1731, 1748-1866, 2791-2941, 3017-3091

Arabidopsis–M1 (4219 aa):– RBG domains (12): 1-149, 150-300, 477-598, 633-780, 942-1159, 1197-1329, 1351-1488, 1533-1712, 1863-1974, 2395-2512, 3436-3565, 3584-3654; accessory domains: PH (801-909), beta-helix (1720-1860), beta-tripod (1983-2152), beta-tripod (2210-2374), C2 (2607-2765), beta-tripod (3972-4220)

Arabidopsis–M2 (4146 aa):– RBG domains (12): 1-169, 170-300, 533-657, 696-839, 1010-1238, 1281-1415, 1444-1573, 1615-1766, 1931-2038, 2440-2560, 3562-3702, 3711-3781; accessory domains: PH (874-989), beta-helix (1790-1930), beta-tripod (2039-2190), beta-tripod (2258-2420), C2 (2660-2820)

Arabidopsis–X (3125 aa):– RBG domains (9): 1-157, 158-314, 465-680, 730-900, 939-1181, 1207-1397, 1611-1742, 2558-2693, 2702-2776; accessory domains: Ricin (1428-1583), helices (partly amphiphathic) (2996-3125)

ATG2

human-B (2078 aa):- RBG domains (8): 1-132,163-335,505-701,738-930,1049-1218,1232-1363,1544-1726,1806-1869

Arabidopsis (1892 aa):- RBG domains (8): 1-120,172-325,457-639,661-869,895-1046,1062-1200,1428-1578,1612-1680

yeast (1592 aa):- RBG domains (8): 1-118,176-338,373-462,492-600,644-756,783-904,1025-1245,1262-1329

SHIP164

human (UHRF1BP1) (1464 aa):- RBG domains (6): 1-105,118-257,349-547,569-831,855-1300,1311-1370

Arabidopsis (1199 aa):- RBG domains (6): 1-126,127-290,315-466,481-654,688-843,902-1068

Hobbit

Human KIAA0100 (Hobbit, 2235 aa):- 1-134, 135-247, 276-393, 415-560, 568-685, 724-846, 869-1113, 1134-1293, 1318-1471, 1574-1774, 1907-2028, 2113-2166; accessory domain: paired helices 884-959, (1429-1444 – *i.e*. missing), 1781-1904

yeast Hob1 (Fmp27, 2628 aa):- RBG domains (12): 1-125, 125-225, 274-426, 467-593, 655-847, 893-1015, 1053-1403, 1429-1651, 1680-1985, 2070-2239, 2340-2470, 2522-2579; accessory domain: paired helices 1067-1234, 1835-1960, 2244-2339

Tweek

yeast Csf1:- RBG domains (17): 61-154, 226-448, 468-607, 627-768, 841-972, 993-1236 (omit loop with 2 helices 1081-1202), 1265-1389, 1534-1610, 1627-1745, 1778-1884, 1920-2035, 2077-2193, 2238-2320, 2354-2472, 2510-2671, 2712-2852, 2891-2922; accessory domains: helical bundle 1410-1520.

Movies

RBG domains only from human VPS13A (N-terminus to the left) showing: Movie 1 back bone only, Movie 2 conserved hydrophilic residues, Movie 3 hydrophobic residues.

Supplementary Table 1:
Homology of RBG domains in VPS13A/B, ATG2B and SHIP164 to the penultimate domains in VPS13 (RBG2 at the N-terminus and RBG11/12 at the C-terminus of VPS13A/B).

RBG domains in the indicated proteins were compared pairwise in HHpred with penultimate domains of VPS13A and VPS13B, as explained in the Methods. pSS >90% = hit (strong); 40-90% = moderate; 10-40% = weak; ≤10% = no hit. "/" indicates no hit or probability shared structure (pSS)<1%. 1st and last RBG domains are included only for VPS13A. * inserts were removed from VPS13A RBG11 (WWE domain) and SHIP164 RBG5 (disordered loop). VPS13C/D follow VPS13A (except for the inserted 3 domains).

A

VPS13		RBG 2 = N-term. (VPS13_N domain)				RBG 11/12 = C-term. (part of Apt1 domain)				notes
		VPS13A		VPS13B		VPS13A*		VPS13B		
	RBG	pSS	cols	pSS	cols	pSS	cols	pSS	cols	
A	1	5	15	7	13	/		/		Chorein_N (100%)
	2	100	156	100	126	6	24	1	24	RBG2
	3	7	38	1	34	99	115	93	68	RBG11/12
	4	97	73	88	53	/		/	23	RBG2
	5	2	44	/		96	120	21	65	RBG11/12
	6	2	17	/		98	115	38	54	RBG11/12
	7	98	126	98	107	/		/		RBG2
	8	1	15	4	38	98	114	97	122	RBG11/12
	9	0	111	40	65	8	111	18	57	RBG2 (mixed)
	10	66	25	77	112	9	27	4	25	moderate RBG2
	11*	3	19	4	28	100	123	100	123	RBG11/12
	12	2	7	/		/		2		VPS13_C
B	2	100	126	100	128	3	22	9	23	RBG2
	3	/		1	50	8	21	15	43	weak RBG11/12
	4	26	51	85	81	/		/		moderate RBG2
	5	35	35	47	108	83	106	81	117	mod. RBG11/12 (mixed)
	6	93	53	96	104	1	17	/		RBG2
	7	2	27	2	35	83	127	80	146	moderate RBG11/12
	8	3	19	5	72	2	48	2	38	none
	9	1	16	1	77	92	102	94	103	RBG11/12
	10	79	56	97	73	2	26	2	76	RBG2
	11	70	51	74	73	/		4	43	moderate RBG2
	12	10	25	7	25	100	127	100	163	RBG11
X	2	91	115	96	114	3	19	6	53	RBG2
	3	/		1	16	28	55	27	62	weak RBG11/12
	4	16	18	40	35	1	16	1	6	weak RBG2
	5	/		/		1	15	/		none
	6	20	53	9	52	2	15	2	15	weak RBG2
	7	1	16	20	43	37	109	31	39	weak RBG11/12 (mixed)
	8	6	23	2	22	99	123	99	125	VPS13_C

B

ATG2B	RBG 2 = N-term. (VPS13_N domain)				RBG 11/12 = C-term. (part of Apt1 domain)				notes
	VPS13A		VPS13B		VPS13A*		VPS13B		
RBG	pSS	cols	pSS	cols	pSS	cols	pSS	cols	
1	14	16	17	15	/		1	6	Chorein_N (100%)
2	98	152	99	126	/		/		RBG2
3	4	18	1	20	16	83	22	46	weak RBG11/12
4	/		9	26	4	41	2	22	none (and variable)
5	/		/		/		/		none
6	34	118	67	116	1	14	3	9	moderate RBG2
7	10	28	15	28	97	106	97	119	RBG11
8	1		1		2	8	/		=V13B RBG13 p94% (68 cols)

C

SHIP164	RBG 2 = N-term. (VPS13_N domain)				RBG 11/12 = C-term. (part of Apt1 domain)				notes
	VPS13A		VPS13B		VPS13A		VPS13B		
RBG	pSS	cols	pSS	cols	pSS	cols	pSS	cols	
1	13	15	19	15	/		/		Chorein_N (100%)
2	97	126	99	125	/		1	21	RBG2
3	1	26	16	79	9	46	3	42	ATG2 RBG7
4	58	54	80	55	/		/		moderate RBG2
5*	/		/		/		/		ATG2 RBG7
6	68	39	87	34	4	14	1	14	moderate RBG2

Supplementary Table 2:
Amphipathic helices immeidately before and after the RBG multimer

Helices predicted by HHpred and/or AlphaFold, not overlapping with TMHs identified by TOPCONS, were tested in Heliquest typically using an 18 mer window, reduced in width when necessary.

A. N-termini

		length	start	end	sample helix	
					hydrophobic face	charge
VPS13	yeast	21	4	24	8/18	0
	human A	21	5	25	8/18	-2
	human B	20	4	23	9/18	+3
	human C	21	5	25	8/18	-2
	human D	21	4	24	8/18	+1
	A. thaliana S	25	3	27	9/18	0
	A. thaliana M1	22	4	25	10/18	+1
	A. thaliana M2	22	4	25	10/18	+1
	A. thaliana X	19	4	22	5/18	+6
ATG2	yeast Atg2	13	7	19	4 / 11	+2
	human A	11	12	22	4 / 11	+2
	human B	13	7	19	4 / 11	+3
	A. thaliana	13	9	21	5 / 11	+1
SHIP164	human	22	2	23	5 / 18	+5
	Dictyostelium	21	2	22	6 / 18	+6
	A. thaliana	22	2	23	7 / 18	+2
Tweek	yeast	25	36	60	6 / 18	+1
	human	16	50	65	7 / 16	+3
	Trichomonas	16	35	50	6 / 16	+2
Hobbit	yeast Fmp27	/				
	yeast Hob2	/				
	human	/				
	A. thaliana	/				

(see over for part B)

Supplementary Table 2 continued:

B. C-termini

		length	start	end	sample helix	
					hydrophobic face	charge
VPS13	yeast	25	2812	2836	5/18	0
	human A	28	2842	2869	5/18	+1.5
	human B	25	3628	3652	8/18	+1
	human C	24	3396	3419	5/18	+1
	human D	23	4044	4066	5/18	-1
	A. thaliana S	20	3088	3107	5/18	+1
	A. thaliana M1	23	3775	3797	5/18	+2
	A. thaliana M2	23	3648	3670	8/18	+1
	A. thaliana X	25	2769	2793	8/18	+3
ATG2	yeast Atg2	18	1315	1332	6/18	0
	human A	18	1721	1738	6/18	0
	human B	18	1864	1881	6/18	0
	A. thaliana	18	1671	1688	7/18	-4
SHIP164	human	/				
	Dictyostelium	/				
	A. thaliana	41	1058	1098	7/18	+1.5
Tweek	yeast	24	2933	2956	7/18	+1
	human	40	4954	4993	7/18	+1.5
	Trichomonas	24	2672	2695	8/18	-1
Hobbit	yeast Fmp27	20	2568	2586	8/18	+3
	yeast Hob2	20	2392	2411	5/18	+1
	human	/				
	Fly	22	2216	2237	5/18	+2
	A. thaliana	18	2407	2424	6/18	+5
